# The COVID-19 Pandemic Revealed the Importance and Shortcomings of Technologies for Diabetes Support

**DOI:** 10.1177/1932296820929721

**Published:** 2020-05-27

**Authors:** Eirik Årsand

**Affiliations:** 1 Department of Computer Science, University of Tromsø – The Arctic University of Norway, Norway; 2 The Norwegian Centre for E-health Research, University Hospital of North Norway, Tromsø, Norway

**Keywords:** COVID-19, diabetes technologies, medication distribution, social media, video conferencing

I have learned that during the COVID-19 pandemic, technologies become more important than ever—especially video conferencing, but also learned that no current health technologies can secure delivery of medicines that keeps those with diabetes alive. I come from Norway, a country that has a fantastic health care system, where—except from a yearly small own share (max. $250)—all medicines and health care is free, for all inhabitants. I have of course read about the difficulties of getting hold of insulin and other medication in United States and other countries, where medicines are extremely costly, and you need to have insurance to receive proper health care. This is hard to understand when always having had all the support you need almost for free. During these times when COVID-19 scares both people and countries to secure themselves first, distribution of medicines and other necessities have shown to be threatened also in my country. This reality really sank in, for many of us I think, during this world-spanning crisis and should encourage us all to work for solid infrastructures for delivery of both medications and health tools for people with serious health conditions.

The COVID-19 pandemic has also demonstrated the potential and importance of personal health technologies. The best example is in my mind the use of video conferencing. We have struggled for decades to make the health care system, both general practitioners and specialists at hospitals, to use video conferencing systems as an alternative and supplementary way of enabling patients to meet health care personnel (HCP). In rural parts of Norway, and I guess many other places, patients may use a whole day or more to travel back and forth to meet their HCP. Then suddenly, the COVID-19 crisis lead to a situation where video conferencing is the only way of facilitating many of the meetings between patients and the HCP. Consequently, in a few days, most are using video conferencing to meet their patients. They do not only use it, they report video conferencing to be a good tool, and a good way to meet!

Most of us have watched, or been part of, the huge increase in health-related social media communities the last few years. We have been fascinated by how people with diabetes organize themselves in various networks, helping each other with both technical challenges and more personal challenges. Even making new functionalities by both programming new software and making hardware in do-it-yourself social media groups. They mark their posts with #WeAreNotWaiting and other patient-empowermental tags. During this COVID-19 pandemic we see how useful these patient groups are in helping and reassuring each other with challenging issues. Much of the current discussions related to COVID-19 I have observed is to interpret and explain the governmental and health authorities’ advices to patients in risk groups like us with diabetes. This is an indication that these advices are not clear enough, and/or not easy enough formulated.

In the future I predict considerable progress in the areas described above, that is, access to medication, use of video conferencing as part of virtual diabetes clinics, and more use of social media. An enhanced effect we can hope for, builds on the fact that patients and people in general use more health sensors than ever, collecting relevant data for health purposes. If we would be able to comprehensively use these, giving both the patients and the HCP access to information derived from analysis helped by algorithms, machine learning, and artificial intelligence, virtual meetings using video conferencing could be really powerful and effective. Today we have continuous glucose monitors, physical activity monitors, insulin pumps, and pens that are able to collect and share data, but the problem is that these are for the most part sent to different servers due to the vendors’ “silo-approach,” making it difficult or impossible to analyze these data together in an efficient way. This needs to be resolved to make the best services and help for the patients.

As we all know, people quickly forget the struggles when things are fixed, but many predict that COVID-19 will be affecting us for many years to come. This sounds like a negative thing; however, it might enable and encourage us to continue the work toward better routines, better tools, and better use of technologies in the time to come. And hinder us from just slipping back to the previous time where “good enough” was enough.

